# What senior academics can do to support reproducible and open research: a short, three-step guide

**DOI:** 10.1186/s13104-022-05999-0

**Published:** 2022-03-22

**Authors:** Olivia S. Kowalczyk, Alexandra Lautarescu, Elisabet Blok, Lorenza Dall’Aglio, Samuel J. Westwood

**Affiliations:** 1 Department of Neuroimaging, Institute of Psychiatry, Psychology and Neuroscience, King’s College London, London, UK; 2 Forensic and Neurodevelopmental Sciences, Institute of Psychiatry, Psychology, Neuroscience, King’s College London, London, UK; 3 Department of Perinatal Imaging and Health, Centre for the Developing Brain, School of Biomedical Imaging and Medical Sciences, King’s College London, London, UK; 4 Department of Child and Adolescent Psychiatry/Psychology, Erasmus MC-Sophia Children’s Hospital, University Medical Centre Rotterdam, Rotterdam, The Netherlands; 5 The Generation R Study Group, Erasmus MC, University Medical Centre Rotterdam, Rotterdam, The Netherlands; 6 Institute of Psychiatry, Psychology, Neuroscience, King’s College London, London, UK; 7 Department of Psychology, School of Social Science, University of Westminster, 115 New Cavendish Street, London, W1W 6UW UK

**Keywords:** Reproducibility, Replication, Authorship, Funding, Publishing, Reform

## Abstract

Increasingly, policies are being introduced to reward and recognise open research practices, while the adoption of such practices into research routines is being facilitated by many grassroots initiatives. However, despite this widespread endorsement and support, as well as various efforts led by early career researchers, open research is yet to be widely adopted. For open research to become the norm, initiatives should engage academics from all career stages, particularly senior academics (namely senior lecturers, readers, professors) given their routine involvement in determining the quality of research. Senior academics, however, face unique challenges in implementing policy changes and supporting grassroots initiatives. Given that—like all researchers—senior academics are motivated by self-interest, this paper lays out three feasible steps that senior academics can take to improve the quality and productivity of their research, that also serve to engender open research. These steps include changing (a) hiring criteria, (b) how scholarly outputs are credited, and (c) how we fund and publish in line with open research principles. The guidance we provide is accompanied by material for further reading.

**Supplementary Information:**

The online version contains supplementary material available at 10.1186/s13104-022-05999-0.

## Introduction

Increasing evidence shows that research in the biomedical and social sciences and research more broadly is difficult to replicate and/or reproduce [1–5]. One of the causes of this ‘replication crisis’ is thought to be misplaced incentives that can undermine research quality. For instance, publishers and funders generally give a selective advantage to novel or statistically significant results, thereby devaluing efforts to confirm published research [6, 7]. Further, employment evaluation criteria unduly focus on individual achievement, publication track records, and grant funding acquisition, which can hamper data sharing and collegiality while incentivising publishing in quantity at the cost to quality [8–11]. Many and varied changes in policies and procedures are seeking to realign incentives to reward transparent, accessible, and reproducible research [12–14], while grassroots initiatives are removing barriers to entry in learning and adopting best research practice [1, 15–24]. However, despite significant support, widespread adoption of open and reproducible research remains elusive [25–28]. Further, there is little attention paid to how the current research culture contributes to bullying, harassment, mental health, and the resulting rising tide of researchers leaving academia [29].

For open research to become the norm, further engagement and support must come from senior academics given their routine involvement in supervision, peer review, journal editing, hiring, and informing institutional policies. Senior academics are, however, presented with unique social and practical barriers. For example, setting higher quality standards for junior researchers can be negatively perceived as ‘ladder pulling’ [30], while the widely held perception that open research can stifle innovation or long-held academic freedoms can make researchers at all career stages hesitant to change current practices [27, 31–33]. Further, applying for grants [34–36] and teaching [37] occupy an increasing amount of work time, which means attending training, developing open research practices, or changing long-standing research routines can be costly and therefore deprioritized. Finally, the increasing literature on how to adopt open research is fast becoming overwhelming, contradictory, and mainly tailored to early career researchers [18, 23, 25, 26].

Therefore, we present a short guide highlighting three easy steps to introduce open research ideas and practices into existing research routines while avoiding the barriers mentioned above. These steps include (1) modifying hiring criteria, (2) crediting scholarly outputs with the contributorship model, and (3) securing grant funding and publishing in line with open research. Following the lead of similar initiatives, these steps are designed to appeal to the self-interests of researchers to motivate their engagement with open research practices [23, 38, 39], with a unique focus on the viewpoint of senior academics. This is supplemented by materials for further reading.

## Main text

### Step 1: Change how you hire

Evidence shows that open research practices confer a competitive advantage in publishing scholarly outputs and acquiring grant funding (see Table 1), meaning that individuals with open research expertise are a desirable asset in lab groups or departments. However, such individuals will likely be missed in hiring and promotion opportunities as a result of the undue weight given to evaluation metrics such as h-indices and journal impact factors [9, 40]. Further, as open research is rarely mentioned in job descriptions, sought-after candidates cannot easily identify potential employers that value open research. Therefore, we encourage senior academics (where possible) to modify their hiring criteria to incorporate open research practices  that support research quality and productivity.

**Table 1:** Open research practices and the career benefits they confer. Definitions are lifted from [43]

Open research practice	Definition	Competitive advantages
Open Access Publishing	A scholarly output accessible to the public free of charge. This can include green, gold or platinum/diamond forms of open access. Open access can be applied to the following scholarly outputs: peer-reviewed journal articles, conference papers, theses, book chapters, monographs, and images	Publishing via open access is associated with higher citation rates and improves the speed and breadth of dissemination of scholarly outputs [44, 45]
Open Data	Publicly accessible, digitally-shareable data that are necessary to reproduce the reported results	Facilitates collaboration [46]; increases efficiency and sustainability [47]; published papers linked with open data and/or materials are associated with a higher citation rate on average [23, 45, 48]; when published with a digital object identifier (DOI), open data and/or materials can be a citable publication [49]; synthetic datasets can help cross-validate analysis and improve reproducibility of analysis workflows [50]
Open Materials	Publicly available components of the research methodology needed to reproduce the reported procedure and analysis (e.g., code, software, workflows, etc.)	
Open Peer Review	A findable, freely and publicly accessible, and signed peer review either pre- or post-publication	Academics who act as reviewers can get credit for their work [51]
Preprints	Complete, non-peer-reviewed manuscript entered in a time-stamped and publicly accessible location, usually an institutional or disciplinary repository (e.g., PsyArXiv, LawArXiv, UCL Press, MedrXiv). Preprints are often also submitted for peer review and publication in a traditional scholarly journal, but this is not mandatory	Wider, faster, and cheaper dissemination of research [52]; greater opportunity for feedback outside of formal peer-review [24]; posting a manuscript as a preprint before formal publication can increase citations and impact [53, 54]; improves chances of publication in journals with high impact factors [55]
Preregistration	A publicly available time-stamped study design and/or analysis plan that is registered in an institutional registration system (e.g., ClinicalTrials.gov, Open Science Framework, AEA Registry, EGAP)	Boost a researcher’s reputation [56]; preventative measure against post-hoc critique (i.e., CARKing—critiquing after the results are known) during peer-review [39, 57, 58]; prospective registration of a study design can be a citable publication; comply with submissions guidelines set by International Committee of Medical Journal Editors (ICMJE)
Registered Reports	A peer-reviewed journal article where the decision to publish is based on a two-stage peer-review process. First, following successful peer-review, a pre-specified study and/or analysis protocol is accepted in principle by a participating journal before data has been collected or accessed. Second, providing the authors closely followed the protocol and successful peer-review, the final manuscript is published regardless of the results	Guaranteed publication regardless of study results, providing the registered protocol and/or analysis is followed [59]; reduces CARKing [39, 57, 58]; cited at comparable or slightly higher levels than conventional peer-reviewed articles [60]; stage one peer-review provides additional peer-review feedback

Modelled on a crowd-sourced initiative [41], one feasible approach is to modify desirable/essential person specification criteria to include a track record of one or more open research practices (e.g., open data, open materials/code, pre-registration, open access publication, publishing preprints, and/or open peer review; see Table 1 for definitions). Criteria should be stated clearly and publicly in advertised job descriptions and/or hiring policies, while decisions about which open research practices to include should be made in consultation with faculties/departments to avoid unnecessarily disadvantaging staff/students. For example, where a track record of open access publications is not expected (e.g., for a PhD student/postdoctoral researcher), proxies for productivity or keen engagement in open research can include preprints, open materials, or open peer review. Instructive examples of how this can be achieved can be found here [42] and in our Additional file 1: Table S1.

### Step 2: Change authorship to contributorship

The main currency for career progression is authorship on scholarly outputs [11, 61, 62]. As a result, authorship disputes are widespread, leading to delays in submissions, conflicts among collaborators and journal editors [63–65], and/or retractions [66–68]. Such intense competition over credit for scholarly outputs has significantly disadvantaged those in more precarious positions (such as black and minority ethnic groups, individuals on fixed-term contracts, and women), with 40% of early-career researchers reporting that credit for their work was given to other academics or research staff [29, 69, 70]. As large collaborative projects become the norm, contributions will be more difficult to dissect and therefore authorship-related issues will become more common [71–74].

Issues with assigning credit for scholarly outputs are in part due to the lack of consensus-based and comprehensive standards. The commonly used standard, the International Committee of Medical Journal Editors (ICMJE; or the Vancouver guidelines), stipulates that authorship is contingent on substantive contributions (e.g., conceptual design, data collection, analysis, or interpretation, drafting and/or revising a manuscript) [75]. Still, ICMJE offers no adequate guidance on contentious issues, such as designating first, last, or corresponding authorship; assigning responsibility for the research; or dealing with large collaborations or other contributions (such as from librarians and statisticians) [76, 77]. These issues can be avoided with contributorship models of authorship, such as the Contributor Roles Taxonomy (CRediT), a consensus-based classification system that distinguishes 14 contributor roles (see Additional file 1: Table S2) that is now adopted in the submission process at leading publishers (e.g., Elsevier, PLoS, Wiley, and Springer) and hundreds of journals [78, 79].

CRediT documents individual contributions to a scholarly output in a standardised, accessible, and discoverable manner. This can be done at any stage in a research project, although the earlier the better to manage expectations of team members and to minimise future authorship issues. The web-based application, *Tenzing*, automates this process and produces a CRediT-compatible manuscript for publication [80]. Although the contributor roles are fixed, their definitions can be customised to a particular research discipline for clarity. Further, CRediT can provide a useful framework for deciding on authorship designation. For instance, the degree of contribution can be specified as ‘lead’, ‘equal’, or ‘supporting’, which can inform authorship order [71, 73]. Moreover, contributions to ‘data curation’, ‘project administration’, and ‘validation’ can instruct who should be the corresponding author. CRediT also offers unique opportunities to improve productivity, particularly in terms of fostering collaborations, by signalling the expertise of members of your research group, recognising individual contributions to large teams, and acknowledging roles which tend to be overlooked despite providing valuable insight or support (e.g., project administration). See Additional file 1: Table S3.

### Step 3: Change how you fund and publish with open research

Funders and journals are beginning to advantage open research practices with novel initiatives and policy changes. Thus, to be in a position of strength, senior academics should engage with open research in seeking funding and publishing their research outputs.

#### Policy changes

Funders and journals widely endorse the practice of making sure that research data should be ‘as open as possible, as closed as necessary’, with new policies being introduced to further compliance with this practice [81]. Most funders now also require a data management plan (i.e., a detailed specification of how data or materials will be curated, shared, or used) as standard [82]. Data availability statements, indicating where data and materials are available or specifying reasons for exemptions from data-sharing, are also compulsory for submissions to a growing number of journals, including Science, Nature, and the BMJ [83–85]. Data can also be archived and shared through data journals (such as, [84–90]) or in third-party repositories (e.g., GitHub, Open Science Framework, and Zenodo), which allow control over how data and code are used and shared by assigning licences and DOIs [1, 49, 93] (See Additional file 1: Table S4).

Perhaps the most significant and less well-known policy changes concern preprints, which encourage the publication of scholarly outputs in a faster, more impactful, and more accessible manner. A preprint is a time-stamped, non-peer reviewed manuscript made freely and publicly accessible via an online server typically within 72-h of submission (e.g., PsyArXiv, LawArXiv). Thus, the significant time lag between manuscript submission and its publication (median days, 165) [94] and the infeasible journal open access fees [95] do not apply to preprints. Because of faster and wider dissemination, grantees are increasingly required to deposit preprints, particularly if funded research is of significant public health benefit (e.g., Bill & Melinda Gates Foundation) [96]. Further, a majority of journals permit preprints to be shared before or during manuscript submission [97] (Additional file 1: Table S4), presumably due to evidence that journal articles linked to preprints have higher citation rates [53, 55]. Influential journals (e.g., BMJ, The Lancet) and funders (e.g., The National Institutes of Health, Wellcome Trust) are now explicitly stating that preprints can be cited [98, 99]. Preprints can additionally be referenced in researcher track records when applying for funding [96] and included in submissions to the UK Government funding organisation, the Research Excellence Framework [98].

#### Funding opportunities

The move from funders to investing in open research is set to gather pace, particularly following the invaluable role open research played in the COVID-19 pandemic [100]. However, identifying and keeping track of open research funding opportunities is challenging. We therefore provided key examples of funding opportunities supporting open research in Table 2 and additionally curated a list of funding opportunities obtained by using data scraping, available at https://lorenzada.github.io/openresearch_funding/. In this list, we selected funding opportunities mentioning keywords related to open research (e.g., replication study, reproducible code, preprint), after data scraping was performed from the NIH and UKRI funding websites. Of note, website selection for data scraping was based on whether automated data collection was permitted for a given website. For further information, please refer to the open code at https://github.com/LorenzaDA/openresearch_funding. This list not only illustrates the mounting financial commitment to open research practices and projects from grant funders, but will hopefully encourage senior academics to apply for funding or for them to support applications from early career researchers in their research team.

**Table 2:** Examples of funding opportunities supporting or rewarding open research, with accompanying text lifted directly from funders’ websites

Funder	Scope
Centre for Open Science	In 2015, the Incubator and Integration Grants provided funding for advancing openness, integrity, and reproducibility in science. Incubator grants supported the development of new open tools and services. Integration grants supported integrating tools and services that are useful to scientists through the Open Science Framework, a free, open-source infrastructure (total budget $300,000) [101] Up to 2019, as part of the Preregistration Challenge, prizes were awarded to researchers who published the results of a preregistered study ($1,000) [102]
The Dutch Research Council (NWO)	Open Science Fund: Grant offering funding to develop, test, and implement novel ways to make science more open, accessible, transparent, and reusable. (up to €50,000) Up until 2019. Replication Studies Grants were offered for replication of existing data (reproducibility), replication with new data, and replication of research questions (total budget €3 million) [103]
The Einstein Foundation Award	The Einstein Foundation Award for Promoting Quality in Research aims to provide recognition and publicity for outstanding efforts that enhance the rigor, reliability, robustness, and transparency of research in the natural sciences, the social sciences, and the humanities, and stimulate awareness and activities fostering research quality among scientists, institutions, funders, and politicians (up to €200,000) [104]
Fostering Responsible Research Practices	Up until 2020, ‘research on research’ funds were awarded to address the need for greater quality, integrity and efficiency in academic research (€75,000 Euro each) [105]
Horizon Europe	Several grant opportunities funded by the European Commission (EU Budget for the Future) for research performed with open science practices and published open access (total budget €95.5 billion) [106]
Learned Societies	Learned societies have also started to reward open research practices. A few notable examples include the British Neuroscience Association Credibility Prize to reward efforts to ensure neuroscience research is as robust, reliable, replicable, and reproducible as possible (£500), and the Organisation for Human Brain Mapping Open Science Award to recognise sustained and impactful efforts in the area of open science ($2500) [107, 108]
Leamer-Rosenthal Prizes	Up until 2017, this prize rewarded social scientists for open research practices (up to $60,000) [109]
Mozilla	Up until 2019. Open Science Mini-Grants provided funding for researchers who are making science more accessible, transparent, and reproducible ($3000–$10,000) [110]
National Institutes of Health (NIH)	A series funding opportunities for creating rigor and reproducibility across several disciplines. Supports open access publication and requires the use of a data management and sharing plan for all grant submissions [111]
National Science Foundation (NSF)	Grant for Ethical and Responsible Research to produce knowledge about what constitutes or promotes responsible or irresponsible conduct of research and why, as well as how to best instil this knowledge into researchers, practitioners, and educators at all career stages (up to $700,000) [112]
QUEST	The QUEST Null Results and Replication Study Award is offering a research bonus to researchers who publish a null result, perform a replication study, preregister a study protocol for a preclinical study, reuse data, or include public engagement in their study (€1,000) [113]
Shuttleworth Foundation Fellowship Programme	Funding for researchers working openly on diverse problems (up to $250,000) [114]
Universities	Universities have started to reward open research practices through Open Research Awards. A few notable examples include the Finnish Open Science Awards, University of Bristol, University of Reading, University of Surrey, University of Groningen. Senior academics can follow this guide to run awards at their own institutions (https://osf.io/kqgez/)
UK Research and Innovation (UKRI)	Provides open-access block grants to enable grant-holders to publish open access [115]
Wellcome Trust	Research Enrichment Fund to support grantholders to use public insights to develop their research (£10,000–250,000) [116] Wellcome Data Re-use prizes to stimulate and celebrate the innovative re-use of research data (£5,000–£15,000) [117] Up until 2021, The Open Research Fund supported individuals and teams anywhere in the world to carry out groundbreaking experiments in open research (£50,000)

### Outlook

‘We create our culture, invisible though it may be, and we therefore have it collectively within ourselves to change our culture for the better’ ([118], p. 92).

Academic researchers typically aim to reach the highest standards of best research practice, but are hampered by perverse incentives and cultural norms. However, senior academics in particular face additional, unique challenges–especially in terms of prohibitive workloads–that prevent them from supporting or practising open research even though they might view open research as necessary or worthwhile. This is a problem. The success of policies and grassroots initiatives aiming to  normalise open research relies on the collective action of researchers, but only when open research is practised routinely by those in positions of seniority can a positive change in research culture and quality take effect. In this context, we sought to lower barriers of entry into open research for senior academics, and to highlight that open research is advantageous for research grant capture, productivity, and integrity. More remains to be done, but our short, easy-to-follow, three-step guide will hopefully mark the first steps into a wider adoption of open research for many senior academics.

## Supplementary Information

**Additional file 1: Table S1**. Examples of open science practices in university policies for hiring and promotion. **Table S2**. The CRediT Taxonomy of Roles (adapted from 71 in main text). **Table S3** Prospective benefits of CRediT (adapted from 71 in main text). **Table S4** List of useful online resources to track funding and journal policies regarding open access, preprints, and open data/materials.

## Data Availability

Not applicable.
