# Supplementary material for: What senior academics can do to support reproducible and open research: a short, three-step guide
Source: BMC Res Notes. 2022 Mar 22;15:116. doi: 10.1186/s13104-022-05999-0 (PMC8938725; doi:10.1186/s13104-022-05999-0)
Supplement: Supplementary file 1 — Additional file 1: Table S1. Examples of open science practices in university policies for hiring and promotion. Table S2. The CRediT Taxonomy of Roles (adapted from 71 in main text). Table S3 Prospective benefits of CRediT (adapted from 71 in main text). Table S4 List of useful online resources to track funding and journal policies regarding open access, preprints, and open data/materials. [file 13104_2022_5999_MOESM1_ESM.pdf]

Table S1. Examples of open science practices in university policies for hiring and promotion

Country	University	Policies
United Kingdom	Bristol	From 2020-21, progression and promotion criteria include: open access publication, open data and code, open materials, preprints, and preregistration of study protocols. Applicants are required to specify their contribution to multi-author publications, and avoid referring to publication metrics such as Journal Impact Factors.<br><br>In November 2019, the university announced the establishment of a Centre for Academic Research Quality linked to the following new positions: 1 Chair, 6 Research Fellows, 6 PhD studentships.
	Glasgow	Criteria for promotion to Professorship include a commitment to open research practices, including open access publications and "transparency of data, methods, materials, design and analysis, and practices that support replication". Journal impact factors are not considered as part of research quality assessment, applicants for promotion are required to select only 4 of their publications to discuss, and CRediT taxonomy is included in all institutional repositories.
	UCL	Applicants for promotion are required to confirm that their publications are openly accessible. Promotion to above grades 7-10 include the expectation that all research outputs are made available through open access wherever possible.
	Cardiff	The academic promotion procedure makes reference to DORA, and specifies that when assessing research quality, consideration will be given to all outputs including datasets and software, as well as a broad range of impact measures.<br><br>Have asked some candidates applying for positions in psychology to provide a track record of open science methods.
Netherlands*	Utrecht	Adapting tenure and promotion criteria to include openness. The university welcomes open access publications, FAIR and open data, sharing code and software, outreach and public engagement. To provide incentives for staff retention, the university aims to fully implement DORA, and facilitate the reuse of data and code.
	TU Delft	Their 2018-2024 strategic framework suggests that R&O evaluation cycles will include explicit recognition of engagement with open science. The university endorses the use of open science practices, which are part of its mission statement. In the guidelines, they promote open science practices across the planning, conducting, and publishing research stages. These include, for example, open research data management and open access publications.
	Eindhoven	Prospective applicants and employees need to abide by the code of conduct which states that research products as well as the materials used to produce such outputs should be made openly available. Such materials include any

		information needed for intersubjective testing of design results and processes. Research data should be available to colleagues, after publication. Publications methods should be meticulously described to ensure that replication studies could be performed based on such descriptions. Additionally, it does not accept the fabrication and falsification of results as well as unjustified selective reporting. Authorship should be granted to researchers who made a significant contribution to a research study.
	Vrije	Their strategic plan for 2017-2020 states that the university will provide infrastructure for new ways to assess academic values to underpin open research ambitions. Research data will be considered as part of assessment, reward and evaluation systems.
USA	Harvard	The School of Engineering and Applied Sciences recommended that faculty applying for promotion or tenure archive their articles in the university's open repository.
	Oregon	Many job advertisements include the following statement: "Our Department embraces the values of open and reproducible science, and candidates are encouraged to address (in their statements and/or cover letter) how they have pursued and/or plan to pursue these goals in their work."
	Wisconsin-Madison	Policies for post-tenure review include considerations for "open-source databases, online tools … and other networked, digital resources related to scholarship" as evidence of productivity.
	Utah Valley	Interviews include the question: ""Describe any steps you have taken to implement the principles of open science in your research".
	IUPUI	Promotion and tenure criteria specify placing higher value on quality rather than quantity of publications, require applicants to specify their exact contribution to publications, and highlight the importance of open-access publication.
	Southern methodist	Encourages all faculty members to practice open science, including preregistration, data sharing, open scripts. These efforts are viewed favourably as part of the annual review.
Germany	Berlin Charite University Hospital	Requires applicants for professorship to report on their open science and reproducible research activities, as well as their contribution to team science. These requirements are part of the MERIT-Quest criteria , which include open research practices such as: distinguishing between exploratory or confirmatory research, pre-registration, open access/code/materials/data, and publication null results. QUEST (Quality-Ethics-Open-Science-Translation) team members screen applications and provide support to hiring committees, to incentivise researchers to adopt responsible research practices. (Strech et al., 2020).
	LMU Munchen	The Dean of Research has submitted a proposal to include open research principles in decisions regarding hiring and tenure-track. This would include the requirement for candidates to write an open-science statement as part of

		their application process. At the moment, the job advertisements in their department of Psychology state “We support transparent research with open data, open material, and pre-registrations”.
	Cologne	Many job advertisements include the statement: “The Department of Psychology aims for transparent and reproducible research (including Open Data, Open Materials, and Preregistrations). Applicants are asked to illustrate how they have pursued these goals in the past and/or how they plan to do so in the future.
	Bielefeld	The Centre for Cognitive Interaction Technology released an Open Science Manifesto and expects researchers to make their data and methods publicly available after a period of exclusive exploitation for their own research questions.
	Leibniz	Is committed to promoting open science practices. Formed the Leibniz Research Alliance Open Science.<br>Researchers at the Fritz Lipmann Institute are required to submit every paper and doctoral thesis to an outside company for integrity vetting before submission.
Canada	Toronto	Job advertisements include the statement: “Our department embraces the values of open science and strives for replicable and reproducible research. We therefore support transparent research with open data, open material, and pre-registrations. Candidates are asked to describe in what way they have already pursued and/or plan to pursue open science.”
	Montreal Neurological Institute	Declared itself to be a fully open science centre and is actively recruiting scientists who are open science advocates. Formed the Tanenbaum Open Science Institute
Belgium	Liege	Candidates for promotion are only considered if their publications have been included in an open access repository.
	Ghent	Research evaluation is done with the aim of combating questionable research practices. There is a 0 tolerance policy towards data falsification and fabrication. Scientific impact of research is assessed based on FAIR principles.
Finland	Helsinki	Is committed to promoting open research practices, with open science being one of its strategic development areas. Researchers must make publications open access.They describe open science as being the foundation of their research, and a main reason why researchers should choose a career there.
	Tampere	Their 2018 action plan states that the university will ensure that the process of merit and reward supports its commitment to open science. They have extensive open science guidelines for their staff. Their 2030 strategy states that they aim to implement a human resource programme to support the university strategy, which includes integrating the principles of open and responsible science into organizational culture.

	The University of Jyväskylä	Received award from Finnish Open Science and Research Award for the most comprehensive measures for promoting openness and visibility.
Spain	Barcelona	Seeks to provide incentives for its academic community to publish open access

*Research at Dutch Universities is assessed using a Standard Evaluation Protocol. From 2021, this will include Open Science principles.

Other universities that have expressed their commitment to promoting open research practices, but for which we could not find any open-research related policies in their hiring and promotion include: University of Virginia, Arizona State, Indiana Bloomington (USA), University of Eastern Finland, LUT University , Abo Akademi, (Finland).

Table S2. The CRediT Taxonomy of Roles (adapted from 71 in main text)

#	Role	Definition	Authors (initials)
1	Conceptualization	Ideas; formulation or evolution of overarching research goals and aims.	
2	Data curation	Management activities to annotate (produce metadata), scrub data and maintain research data (including software code, where it is necessary for interpreting the data itself) for initial use and later re-use.	
3	Formal analysis	Application of statistical, mathematical, computational, or other formal techniques to analyse or synthesize study data.	
4	Funding acquisition	Acquisition of the financial support for the project leading to this publication.	
5	Investigation	Conducting a research and investigation process, specifically performing the experiments, or data/evidence collection.	
6	Methodology	Development or design of methodology; creation of models.	
7	Project administration	Management and coordination responsibility for the research activity planning and execution.	
8	Resources	Provision of study materials, reagents, materials, patients, laboratory samples, animals, instrumentation, computing resources, or other analysis tools.	
9	Software	Programming, software development; designing computer programs; implementation of the computer code and supporting algorithms; testing of existing code components.	
10	Supervision	Oversight and leadership responsibility for the research activity planning and execution, including mentorship external to the core team.	

Table S3. Prospective benefits of CRediT (adapted from 71 in main text)

- Providing visibility and recognition for researchers working in large teams whose individual contributions are lost in an expansive author list.
- Providing visibility for a diverse range of research contributions that are key to research output being published beyond a traditional focus on writing and drafting (e.g. data curation, statistical analysis, etc.).
- Supporting research institutions and authors to resolve author disputes by providing more transparency around individual author roles and responsibility.
- Supporting research and researcher evaluation by providing a more holistic and nuanced view of the contributions of researchers to research output.
- Improving the ability to track the outputs and contributions of individual research specialists and grant recipients.
- Easy identification of potential collaborators and opportunities for research networking.
- Supporting identification of potential reviewers, experts, and specialists for a variety of roles across research.

Table S4. List of useful online resources to track funding and journal policies regarding open access, preprints, and open data/materials. Accompanying text is lifted directly from the corresponding website.

Resource (URL)	Description
Digital Curation Centre (https://www.dcc.ac.uk/about)	The DCC provides expert advice and practical help on how to store, manage, protect, and share digital research data. They provide a broad range of resources including online tools, guidance, and training. DCC also provides consultancy services on issues such as policy development and data management planning.
FAIRsharing.org (https://fairsharing.org/)	A curated, informative, and educational resource on data and metadata standards, inter-related to databases and data policies.
Sherpa Juliet (https://v2.sherpa.ac.uk/juliet/)	Sherpa Juliet is a searchable database and single focal point of up-to-date information concerning funders' policies and their requirements on open access, publication and data archiving.
Sherpa Romeo (https://v2.sherpa.ac.uk/romeo/)	Sherpa Romeo is an online resource that aggregates and analyses publisher open access policies from around the world and provides summaries of publisher copyright and open access archiving policies on a journal-by-journal basis.
Transparency & Openness Promotion (TOP) Factor (https://www.topfactor.org/)	An alternative to journal impact factor (JIF) to evaluate qualities of journals, the TOP Factor assesses journal policies for the degree to which they promote core scholarly norms of transparency and reproducibility.
Transpose (https://transpose-publishing.github.io/#/)	A database of journal policies on peer review, co-reviewing, and preprinting.
